# Definitions of Computer-Assisted Surgery and Intervention, Image-Guided Surgery and Intervention, Hybrid Operating Room, and Guidance Systems: Strasbourg International Consensus Study

**DOI:** 10.1097/AS9.0000000000000021

**Published:** 2020-11-20

**Authors:** Mariano Giménez, Benôit Gallix, Guido Costamagna, Jean-Nicolas Vauthey, Michael Moche, Go Wakabayashi, Reto Bale, Lee Swanström, Jürgen Futterer, David Geller, Juan M. Verde, Alain García Vazquez, Ivo Boškoski, Nicolas Golse, Beat Müller-Stich, Bernard Dallemagne, Mårten Falkenberg, Sven Jonas, Carina Riediger, Michele Diana, Niklas Kvarnström, Bruno C. Odisio, Edgardo Serra, Christiaan Overduin, Mariano Palermo, Didier Mutter, Silvana Perretta, Patrick Pessaux, Luc Soler, Alexandre Hostettler, Toby Collins, Stéphane Cotin, Michael Kostrzewa, Amilcar Alzaga, Martin Smith, Jacques Marescaux

**Affiliations:** From the * Research Institute against Cancer of the Digestive System (IRCAD), Strasbourg, France; † Institut Hospitalo-Universitaire IHU, University of Strasbourg, Strasbourg, France; ‡ DAICIM Foundation, Buenos Aires, Argentina; § Department of Radiology, McGill University & Health Centre, Quebec, Canada; ∥ Digestive Endoscopy Unit, Fondazione Policlinico Universitario Agostino Gemelli IRCCS, and Centre for Endoscopic Research Therapeutics and Training (CERTT), Università Cattolica del Sacro Cuore, Rome, Italy; ¶ Department of Surgical Oncology, The University of Texas MD Anderson Cancer Center, Houston, TX; # Department of Interventional Radiology, Helios Park-Klinikum, Leipzig, Germany; ** Department of Surgery, Ageo Central General Hospital, Saitama, Japan; †† Interventional Oncology—Microinvasive Therapy (SIP), University Clinic for Radiology, Medical University, Innsbruck, Austria; ‡‡ Radboud University Medical Center, Nijmegen, The Netherlands; §§ Division of Hepatobiliary and Pancreatic Surgery, University of Pittsburgh, Pittsburgh, PA; ∥∥ Centre Hépato-Biliaire, Hôpital Paul Brousse, Villejuif, France; ¶¶ Minimally Invasive Surgery, Surgical Clinic University Hospital, Heidelberg, Germany; ## Service de Chirurgie Digestive & Endocrinienne, Hôpitaux Universitaires de Strasbourg, Strasbourg, France; *** Department of Radiology at the Department of Clinical Sciences, Sahlgrenska University Hospital, Gothenburg, Sweden; ††† Department of Visceral Surgery, 310Klinik, Nuremberg, Germany; ‡‡‡ Department of Visceral, Thoracic and Vascular Surgery, University Hospital Carl Gustav Carus, Technische Universität Dresden, Dresden, Germany; §§§ Department of Surgery at Institute of Clinical Sciences, Sahlgrenska University Hospital, Gothenburg, Sweden; ∥∥∥ Department of Interventional Radiology, The University of Texas MD Anderson Cancer Center, Houston, TX; ¶¶¶ Radboud University Medical Centre, Nijmegen, The Netherlands; ### Visible Patient, Strasbourg, France; **** INRIA, Strasbourg. France; †††† Advanced Therapies Surgery, Siemens, Forchheim, Germany; ‡ ‡‡‡Department of Surgery, University of the Witwatersrand, Johannesburg, South Africa.

**Keywords:** computer assistance, image guidance, guidance systems, hybrid operating room, minimal invasion

## Abstract

To develop consensus definitions of image-guided surgery, computer-assisted surgery, hybrid operating room, and surgical navigation systems.

**Summary Background Data:**

The use of minimally invasive procedures has increased tremendously over the past 2 decades, but terminology related to image-guided minimally invasive procedures has not been standardized, which is a barrier to clear communication.

**Methods:**

Experts in image-guided techniques and specialized engineers were invited to engage in a systematic process to develop consensus definitions of the key terms listed above. The process was designed following review of common consensus-development methodologies and included participation in 4 online surveys and a post-surveys face-to-face panel meeting held in Strasbourg, France.

**Results:**

The experts settled on the terms computer-assisted surgery and intervention, image-guided surgery and intervention, hybrid operating room, and guidance systems and agreed-upon definitions of these terms, with rates of consensus of more than 80% for each term. The methodology used proved to be a compelling strategy to overcome the current difficulties related to data growth rates and technological convergence in this field.

**Conclusions:**

Our multidisciplinary collaborative approach resulted in consensus definitions that may improve communication, knowledge transfer, collaboration, and research in the rapidly changing field of image-guided minimally invasive techniques.

## Abstract 2

This study gathered experts with multiple backgrounds and used common consensus-development methodologies in 2 phases (online and panel meeting). It resulted in definitions that may improve communication, knowledge transfer, collaboration, and research in the field of image-guided minimally invasive techniques.

## INTRODUCTION

The use of minimally invasive procedures has increased tremendously over the past 2 decades. Increasing numbers of professionals have adopted minimally invasive procedures in their clinical practices, including radiologists, surgeons, and endoscopists. Multiple factors have contributed to the development of minimally invasive procedures, including advances in medical imaging, which made it possible for pioneers to begin conducting procedures under “image guidance.”

**FIGURE 1.**
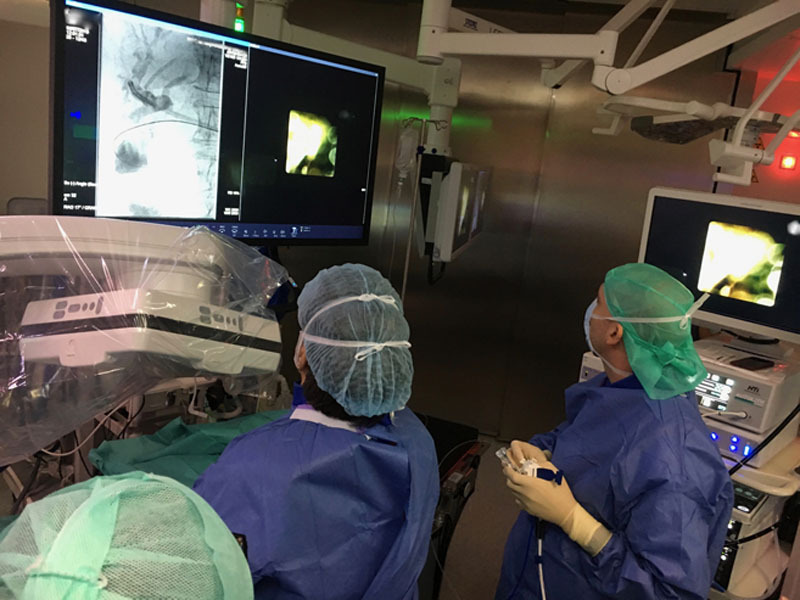
Collaboration between a biliopancreatic endoscopist and an interventional radiologist in the case of a patient with a previous total gastrectomy and large common bile duct stones. A SpyGlass direct visualization system (Boston Scientific, Marlborough, USA) was introduced percutaneously, and electrohydraulic lithotripsy was performed until complete stone clearance. High-definition medical imaging systems (computed tomography, cone beam computed tomography, and magnetic resonance imaging) combined in the same suite allowed the use of real-time and 3-dimensional imaging to show the absence of residual stones (ARTIS Pheno, SOMATOM, and MAGNETOM systems; Siemens Healthineers, Forchheim, Germany).

**FIGURE 2.**
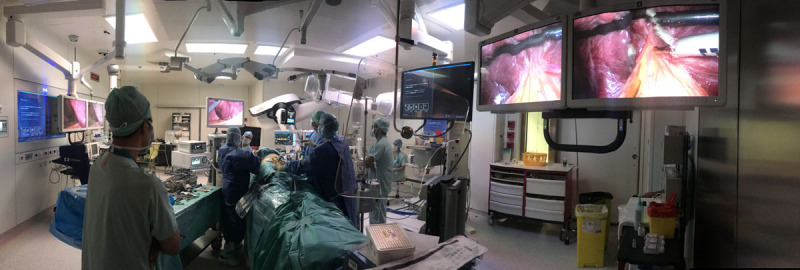
Collaboration in a hybrid operating room between a surgeon and biliopancreatic endoscopists in the case of a patient with gallbladder and common bile duct stones and a Roux-en-Y gastric bypass. A 12-mm trocar was placed in the gastric fundus, and conventional endoscopic retrograde cholangiopancreatography (ERCP) was done, followed by cholecystectomy. In the case of difficult cannulation, a wire could be passed through the cystic duct or percutaneously through the liver into the bile duct to facilitate ERCP.

Image-guided minimally invasive procedures for complex diseases often involve cooperation among specialists from multiple disciplines, for example, surgery, gastrointestinal endoscopy, and interventional radiology.^1–7^ Two of the most common multidisciplinary minimally invasive procedures for complex diseases are lithotripsy (Fig. 1) and endoscopic retrograde cholangiopancreatography and cholecystectomy (Fig. 2). The incorporation of image guidance into these and similar procedures improves understanding of anatomy, facilitates appropriate selection of instruments, and leads to high-quality outcomes with reduced risk of complications.

The visual information obtained during image-guided minimally invasive procedures is 2-dimensional, and as a consequence, better visual data manipulation capacity is required than is necessary with open and laparoscopic surgery. This has opened the door to a great number of technologies implementing input-process-output models to transform huge amounts of data into valuable information. This adoption of “computer assistance” or ”computer-integrated systems” has resulted in exponential growth of human-computer interfaces, with more players (physicians, other caregivers, developers, engineers, etc.) jumping into the field to develop such interfaces and transfer them to the clinic. Specialists are now working with images coming from a number of different fields (eg, gastroenterology, digestive surgery, and interventional radiology).

The increasing interest in image guidance, the heterogeneous backgrounds of the teams working in this area, the enormous amounts of data associated with image guidance, and the growing implementation of image-guidance-related technologies make the field of image-guided minimally invasive procedures extremely dynamic. Consequently, clear communication in this field is exceptionally important. However, the terminology in this field has not been standardized, which is a barrier to clear communication.

Therefore, we undertook a consensus process involving experts in interventional radiology, therapeutic endoscopy, minimally invasive surgery, and information technology to reach agreement about key terms related to image-guided minimally invasive procedures.

## METHODS

### Genesis of the Project and First Steps

We noted that during formal presentations at clinical congresses and less formal discussions between specialists with different backgrounds, terms used to describe minimally invasive procedures were assigned different meanings by different speakers. Recognizing that establishing consistent terminology would facilitate communication, collaboration, and research in this field, we aimed to use a collaborative and co-creative approach to develop consensus definitions for key terms. Two main questions needed to be answered at the outset of the project: in what content areas did terms need to be defined, and what was the best methodology by which to arrive at consensus definitions. The core authors of the consensus document (J.M., M.G., E.S., A.G.V., and J.M.V.) individually identified areas of interest, and then they met for a face-to-face discussion. They agreed upon 5 main content areas: computer-integrated systems, surgical and/or interventional facilities, navigation/guidance systems, artificial intelligence and deep learning, and immersive technologies. The core authors then individually conducted bibliographic research in these 5 areas, without constraints, to determine the state-of-the-art. They tabulated and synthesized their findings and shared them with the other core authors. During this process, the authors confirmed numerous inconsistencies in the definitions of key terms in the 5 main content areas, as well as lack of a common lexicon; they also found that little relevant information was available, and much of it appeared in low-quality publications.

At that point, the core authors decided to start from scratch, abandoning the literature-review approach and instead seeking consensus from a collaboration among experts. The design of the best methodology for this effort was a challenge and required an extensive review of methodologies previous used for consensus development. The methods reviewed included Delphi, modified Delphi, RAND, nominal group process, consensus development panel, and consensus development guided by use of proprietary software.^8–12^ The core authors concluded that no method on its own would be sufficient and that there is no consensus on consensus methodologies.^13^ The Nominal group process does not contemplate a remote phase, the Delphi technique face-to-face interactions, and both consensus development panel and software any kind of private decisions. RAND approach and modified Delphi were limited for the number of participants, and they also require preexistent and consistent definitions.

Next, the core authors meticulously analyzed the most commonly used consensus models and developed a hybrid approach relying on the strengths of different models. The most commonly employed consensus methodologies, nominal group process, consensus development panel, and Delphi technique, were used as templates.^8–12^

#### Recruitment of International Experts

The core authors decided to identify experts in the 5 main content areas and invite them to help develop consensus definitions of key terms. Not only clinical providers but also engineers were invited to participate. An expert was defined as any professional actively involved in image-guided minimally invasive procedures on a daily basis. Experts were identified through online searches, existing relationships, recommendations from colleagues, and contacts with companies developing related products (eg, Siemens). To promote creativity and reproducibility, the core authors decided that 3 or more continents and 10 or more countries should be represented. The identified experts were formally invited to participate by email or phone.

#### Design of Consensus Development Process

The core authors designed a 2-phase consensus development process. Phase 1 was a series of online questionnaires, and phase 2 was a 1-day face-to-face panel meeting.

Because a researcher’s bias could be increased if the researcher also acted as an expert contributor, and because the core authors anticipated that the consensus development process would be time-consuming and demanding, 2 of the 5 core authors (A.G. and J.M.V.) were designated, with their agreement, to act as the researchers overseeing the study. These 2 authors did not participate as experts in the study. Their complete independence and the anonymity and privacy of the online phase were intended to decrease internal and external influences.

Regarding phase 1 (the online phase), the core authors decided that they would:

(1) develop at least 2 surveys (with an expected overall and individual response rate of over 70%) to allow sufficient opportunity to refine the definitions, but limit the total number of surveys to 4 to minimize incomplete responses due to survey fatigue;
(2) begin with a double-blind survey to decrease local bias (same-institution bias) and research bias (external influences), and allow sufficient time for thorough analysis and comprehensive interpretation of the data;
(3) write clear surveys with open-ended answers;
(4) pay special attention to any sign of confusion in the answers and immediately discard any confusing questions;
(5) preserve privacy in subsequent surveys to avoid any kind of expert-expert interaction and thereby decrease expert bias and the “jump on the bandwagon” effect;
(6) move from a qualitative survey at the outset to semi-quantitative surveys later to permit an intermediate consensus agreement to be reached and a good understanding of the results to be achieved before the planned panel meeting; and
(7) set greater than 80% agreement as the threshold used to judge that partial or intermediate consensus had been reached.^13^

Regarding phase 2, the core authors decided to hold a 1-day meeting in Strasbourg, with the morning devoted to presenting and discussing the survey findings and arriving at a consensus among the experts and the afternoon devoted to developing a roadmap for future projects.

#### Survey Structure and Plans for Data Analysis

The first survey comprised 17 open-ended questions (text fields without restrictions) about respondents’ definitions of and views regarding elements of computer-aided surgery, image-guided surgery, guidance systems, hybrid operating room, and immersive technologies and artificial intelligence.

The data from the surveys were analyzed using qualitative word cloud techniques (Python WordCloud library and Python Language Reference, version 2.7^14^).

## RESULTS AND DISCUSSION

The design of the consensus started on February 1, 2019 (day 1), the online phase started on March 1, 2019 (day 29) and ended on May 24, 2019 (day 113), and the meeting in Strasbourg took place on June 13, 2019 (day 133).

### Participating Experts

Thirty-five experts were invited to participate, and 27 agreed, for an acceptance rate of 77%. The participating experts included 6 interventional radiologists, 3 therapeutic endoscopists, 16 surgeons with expertise in percutaneous/minimally invasive surgery, and 2 nonphysician experts (an engineer and a technical physician). The experts were from 10 different countries from 4 continents.

### Online Surveys: Content, Timing, and Participation Rates

During the online phase, 4 surveys were administered using Google Forms (Google 2019 ^15^). With 4 surveys and 27 participating experts, a total of 108 surveys could have been completed; 89 were actually completed, for an overall response rate of 82%. The response rates for the individual surveys ranged from 75% to 93%.

**FIGURE 3.**
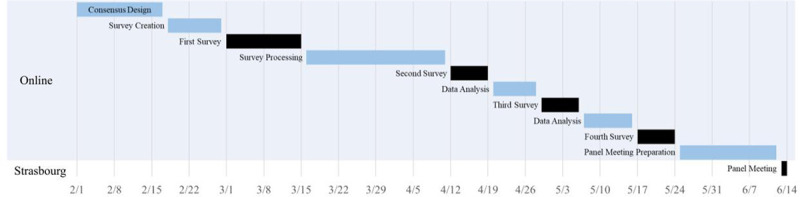
Timing of work in the online-survey phase.

The first survey (anonymous, double-blind) not only was the most difficult to develop, because of the effort required to write clear and precisely focused questions, but also the most difficult to analyze because of the heterogeneous and comprehensive responses. The word cloud analysis was also a useful tool to trigger the brainstorming sessions of the researchers and to report the data during the panel meeting. The total time elapsed for the first survey was 53 days. This included 11 days for survey creation and testing (before the online phase officially began), 15 days when the survey was open for expert contributions (the first of these 15 days marked the beginning of the online phase), and 27 days for analysis of responses (Fig. 3). Following analysis of the survey responses, 1 question that asked the experts to “propose an organization chart” for concepts related to minimally invasive surgery was discarded because inconsistencies in the respondents’ answers indicated that the question was hard to understand.

The researchers decided to narrow the scope of the second survey to 4 areas: computer-assisted surgery, image-guided surgery, hybrid operating room, and navigation systems. For each area, the second survey (single-blind, experts) included a proposed definition, 4 to 6 terms that experts were asked to rate as being essential or not to the definition, and a text field allowing the experts to comment on the proposed definition and propose changes. The survey also included a final question about which of 19 listed actions should be included in a standard workflow for image-guided procedures. Participants could check 1 box, several boxes, or none, and they could write in their own choice. Nine of the 19 attached statements were each checked by more than 50% of respondents. The total time elapsed for the second survey was 44 days. This included 27 days for survey development, 8 days when the survey was open for expert contributions, and 9 days for analysis of responses.

The third and fourth surveys (single-blind, experts) contained different definitions of concepts and asked experts to rate each definition on a Likert scale with 5 options ranging from strongly agree to strongly disagree. The time elapsed for the third and fourth surveys together was 26 days. On the third survey, the mean rate of agreement on the proposed definitions was 85%, and rates of agreement for the individual sections ranged from 88% to 96%. The difference between these 2 surveys was that survey 4 included an addendum to the definitions.

### Panel Meeting in Strasbourg

The Strasbourg International Conference took place on June 13, 2019. A total of 17 experts (63%) attended.

In the morning, the researchers (A.G. and J.M.V.) presented the data and the final version of each definition. This presentation was followed by a question-and-answer session and a panel discussion. A final vote was taken using an in-place polling system to determine the overall degree of consensus achieved. After 1.5 hours of discussion, the proportion of experts agreeing with all 4 definitions was 82.3% with the following minimal modifications in the definition titles: computer-assisted surgery was changed to computer-assisted surgery and intervention, and image-guided surgery was changed to image-guided surgery and intervention.

In the afternoon, the experts were divided into 3 teams to discuss 3 topics. The goal of the first team was to determine whether or not task forces or working groups were needed to keep track of emerging important concerns in the field of image-guided minimally invasive procedures. To kick off the activity, the researchers gave the team 5 possible task forces: industrial liaisons, potential partnerships, education endeavors, research and development, and writing activities. The goal of the second team was to develop the first draft of a common workflow for image-guided minimally invasive procedures that would be comprehensive, reproducible, and scalable. The goal of the third team was to address ethical matters related to new disruptive technologies and new procedures or therapies in image-guided minimally invasive surgery and intervention, trying to detect current and/or potential dilemmas and how to deal with them.

At the end of the afternoon, after the teams had worked for 3 hours, a plenary session took place during which the teams shared their results and gathered feedback. At the end of the plenary session, a poll was conducted, and the results demonstrated full agreement among the expert participants with the proposals made by the 3 teams.

### Final Definitions

For a summary of the definitions see Table 1.

### Image-Guided Surgery and Intervention

**FIGURE 4.**
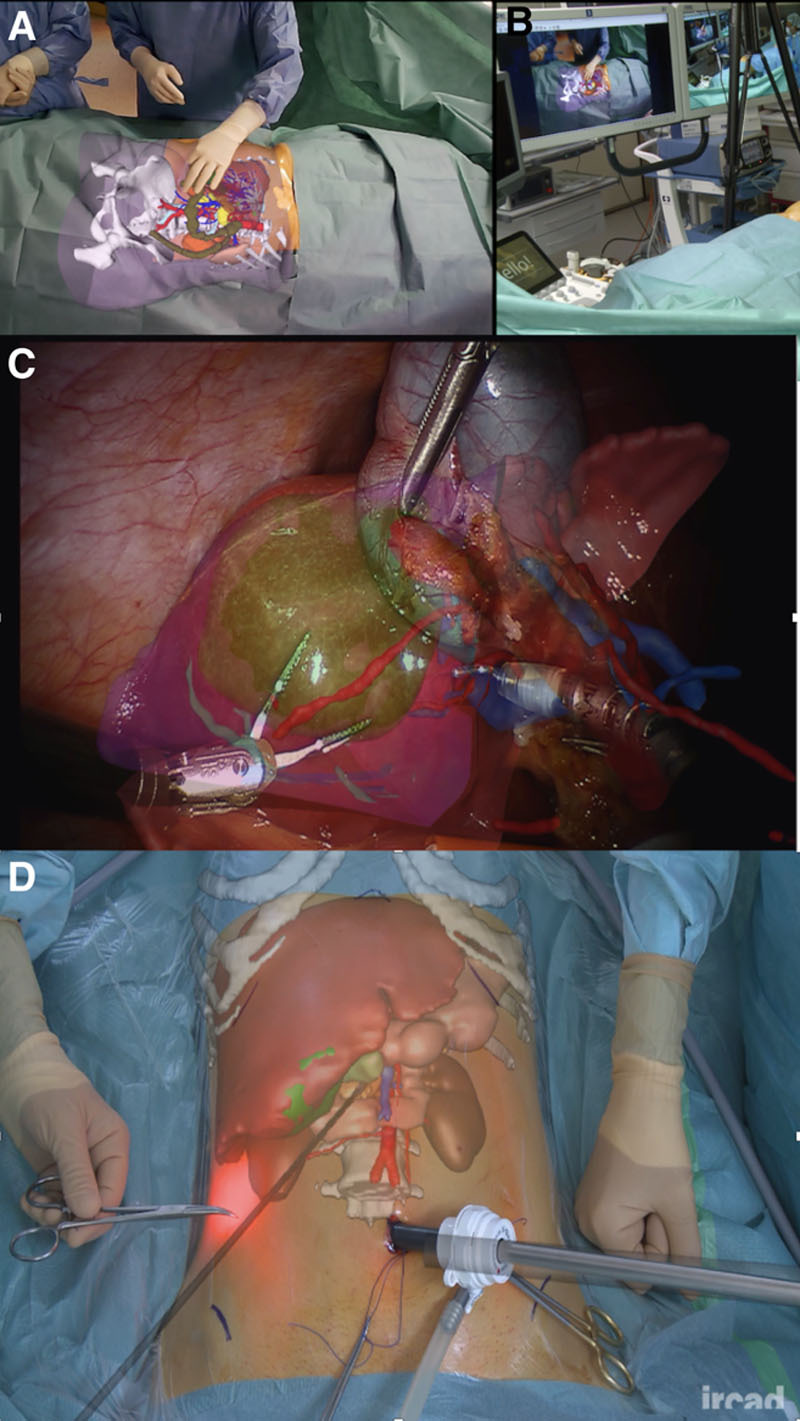
Image-guided surgery and intervention. During the planning phase, a contrast-enhanced computed tomography scan acquisition is post-processed and segmented to obtain the 3-dimensional models employed to provide augmented reality. A and B, Show the use of the visual augmentation assisting the initial phase of a laparoscopic approach. C, Shows the same augmentation overlaid in the display used for laparoscopic vision, and (D) the possible switching from one to the other augmentations throughout the procedure.

As expected, the first survey produced the largest amount of information by far. Analysis of the results showed that procedure, use, specific, technology, and planning were the words most often mentioned. After analyzing the results of the first survey, the researchers attempted to conceive of the field of image guidance as a discipline, a specialty, or maybe a new set of skills (radiology, surgery, endoscopy) or to view this field in terms of imaging methods (radiography, CT, MR, etc.); however, these attempts were not successful. The researchers ultimately decided to understand image guidance as the incorporation of imaging as an integral element of the minimally invasive procedure. The survey responses also indicated that collaboration between different disciplines and simultaneous convergence of technologies can disrupt this field. Consequently, the researchers proposed the following definition of image-guided surgery reflecting collaboration between professionals, convergence of disruptive technologies, and their integration at the center of image-guided minimally invasive techniques (Fig. 4):

The synergy between interdisciplinary collaboration and convergence of multiple technologies (eg, guidance systems, immersive technologies), providing extensive visual information layers (eg, spectrum, resolution, transparency) and making them intuitive, upgrading existing surgical skills and forging new ones. Due to its comprehensive mindset (planning, guidance, control), a breakthrough transformation emerges to enforce state-of-the-art procedures and develop others, thereby achieving precision.

Between the third and fourth surveys, the researchers changed the title of this item from “image-guided surgery” to “image-guided surgery and intervention,” and as a result, the proportion of experts agreeing with the definition changed from 69% in the third round to 88% in the fourth round.

Both in responses to the online surveys and in face-to-face meetings, participants described image guidance as an evolution of other minimally invasive techniques, sharing common roots with interventional radiology, therapeutic endoscopy, and minimally invasive surgery. Likewise, the surveys showed that imaging methods relying on coordinate systems are used to guide procedures and provide a better understanding of anatomy to prevent damage to neighboring structures (by increasing situational awareness) but also increase exposure to ionizing radiation. Thus, strict adherence to radiation protection guidelines, such as those published by the relevant European and American societies,^16,17^ is crucial.

### Computer-Assisted Surgery and Intervention

Because respondents to the first survey seemed to use computer assistance as a synonym for or as a part of image guidance, the researchers decided to dedicate a separate section on the second survey to computer assistance. Surprisingly, none of the responses to the second survey reinforced the idea that computer assistance is part of image guidance. Word cloud analysis showed that images and imaging were not among the top words associated with computer assistance and that the words most commonly associated with computer assistance were technology, planning, and procedures. After a detailed word-by-word discussion of the information collected from the first survey, the researchers proposed defining computer-assisted surgery as a way to enforce the skills of the physician but also to augment them, providing abilities that cannot be acquired without these tools:

Broad use of information technology frameworks to enhance physicians’ skills and augment senses (eg, image-guided surgery), cognition (eg, deep learning, machine learning), and execution (eg, mechatronic, imaging and surgical robotics) with the aim to provide more precise and safer procedures.

This definition of computer assistance was widely agreed upon, by 90% of experts in the third survey and 91.5% in the fourth survey after minor modifications, the most important of which was the addition of and intervention in the title of the definition. One important contribution worth mention is the role of image post-processing (eg, 3-dimensional modeling) and immersive technologies (augmented, mixed, and virtual reality), providing user-friendly human-machine interfaces and making real-time information management easier and more intuitive. Even though these advanced tools can improve the operator’s skills, a minimum required expertise in image reading should be mandatory before any procedure is started. Also related, a wide range of extra computer-assisted tools (3-dimensional modeling, simulations, etc.) can be integrated into training activities to help providers improve their abilities and learn new surgical skills before taking care of the patient.

### Guidance Systems

In the first survey, the term *navigation systems* was used. After reviewing the results of that survey and conducting a deep dive into the field, and relying on solid concepts and definitions coming from different partners,^18^ the researchers proposed changing the broad term to *guidance systems* and conceptualizing navigation as a key feature of guidance systems. Ultimately, a definition was settled on in which guidance systems are conceptualized as having 3 core elements: guidance (assessing the vector from origin to target), navigation (information about the track from origin to target), and control (dynamic modification capabilities):

Any technology combining 3 core elements (guidance, navigation, and control), bringing location data and improving spatial orientation at any time during the procedure, making it possible to reach targets with increased precision and minimal disruption to surrounding tissues.

Among other evolving technologies, these systems need to grow associated in conjunction with visual data (eg, medical imaging), developing intuitive human-machine interfaces, and facilitating the planning strategy and tracking the position of instruments throughout the procedure.

This definition was widely accepted, with 85% and 96% acceptance rates in the third and fourth surveys, respectively.

### Hybrid Operating Room

**FIGURE 5.**
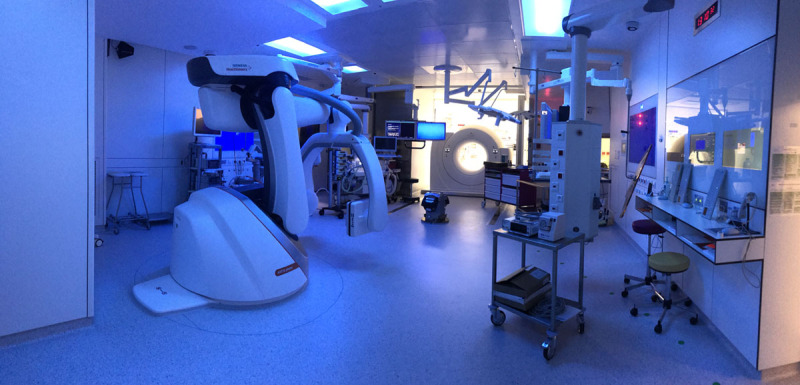
Photograph of a hybrid operating room. In this case, computed tomography, magnetic resonance imaging, and cone-beam computed tomography systems (Siemens Healthineers, Forchheim, Germany) are combined in the same suite.

After the systematic approach previously described, the rough idea of an advanced surgical and imaging facility was established, and the next step to complete the definition was related to imaging techniques and their role in the surgical/interventional setting (Fig. 5). The following definition of a hybrid operating room was proposed:

Facility equipped with full surgical capabilities, including medical imaging based on coordinate systems (CT, MR, cone-beam CT) associated with other techniques (ultrasound, fluoroscopy) and/or guidance systems. Through different types of human-machine interfaces, the planning, guidance, and control stages can be performed intraoperatively in a dynamic fashion.

After the third and fourth online surveys, on which 95% and 96% of the respondents, respectively, agreed with the above definition, most of the experts expressed the need for a hybrid operating room classification. After the first 2 rounds, and after an initial attempt to classify hybrid operating rooms according to the number of imaging technologies was rejected, the classification detailed in Table 2 was agreed upon. These suites were conceived of as the field where multiple types of information need to be put together in a comprehensive way, and an input-process-output approach was used to determine and separate different types of set-ups. This classification considers the facility input, its interfaces (processes), and usage essential levels (output) and stratifies their levels from 1 to 4 depending on task complexities.

**TABLE 1.** Summary of the Definitions

Image-guided surgery and intervention
The synergy between interdisciplinary collaboration and convergence of multiple technologies (eg, guidance systems, immersive technologies), providing extensive visual information layers (eg, spectrum, resolution, transparency) and making them intuitive, upgrading existing surgical skills and forging new ones. Due to its comprehensive mindset (planning, guidance, control), a breakthrough transformation emerges to enforce state-of-the-art procedures and develop others, thereby achieving precision
Computer-assisted surgery and intervention
Broad use of information technology frameworks to enhance physicians’ skills and augment senses (eg, image-guided surgery), cognition (eg, deep learning, machine learning), and execution (eg, mechatronic, imaging and surgical robotics) with the aim to provide more precise and safer procedures
Guidance systems
Any technology combining 3 core elements (guidance, navigation, and control), bringing location data and improving spatial orientation at any time during the procedure, making it possible to reach targets with increased precision and minimal disruption to surrounding tissues. Among other evolving technologies, these systems need to grow associated in conjunction with visual data (eg, medical imaging), developing intuitive human-machine interfaces, and facilitating the planning strategy and tracking the position of instruments throughout the procedure
Hybrid operating room
Facility equipped with full surgical capabilities, including medical imaging based on coordinate systems (CT, MR, cone-beam CT) associated with other techniques (ultrasound, fluoroscopy) and/or guidance systems. Through different types of human-machine interfaces, the planning, guidance, and control stages can be performed intraoperatively in a dynamic fashion

**TABLE 2.** Classification of Hybrid Operating Room Complexity

Scoring of Input, Human-Machine Interface, and Usage			
Characteristic	Essential Elements (AND/OR)		
Input (I)	CT	MR	CBCT
Human-machine interface (H)	Basic features*	Advanced features†	
Usage (U)	Prepared for open surgery	Surgical robot	Imaging robot
Definitions of HOR complexity levels			
Note that at least 1 essential element should be present in each category to be considered an HOR			
Level 1 = only 1 essential element per characteristic (eg, I_1_H_1_U_1_ means that only one essential element is present in the HOR)			
Level 2 = 1 characteristic with more than one essential element (eg, I_2_H_1_U_1_ if the HOR has both CT and MR capabilities)			
Level 3 = 2 characteristic with more than one essential element (eg, I_2_H_2_U_1_ if the previous HOR adds basic and advanced human-interface features)			
Level 4 = 3 characteristic with more than one essential element (eg, I_2_H_2_U_2_ if the previous HOR is prepared for open surgery and includes a surgical robot)			

*Basic features: measuring and planning tools.

†Advanced features: image fusion or augmentation.

CBCT, cone beam CT; HOR, hybrid operating room.

## LIMITATIONS

The present study has 2 main limitations. The first is related to the design of the consensus, which combines features of different validated methods, and this was done because no method on its own would be sufficient, contemplating remote and face-to-face stages, a numerous group of participants, and the lack of preexistent definitions. The second limitation is the fact that the experts participating were from a variety of specialties including physicians, engineers and technicians coming from healthcare facilities, researchers and industry, because the authors felt that the discussion around introduction of new technologies in the operating room should include multiple profiles and backgrounds. This led to a rather heterogeneous group for the 2 phases of the consensus which might have affected the outcomes of the voting. This was done because of the technical and investigational subject matter being looked at and it was felt that a broad representation of the stakeholders involved would yield a more balanced end result.

## CONCLUSION AND FUTURE DIRECTIONS

**FIGURE 6.**
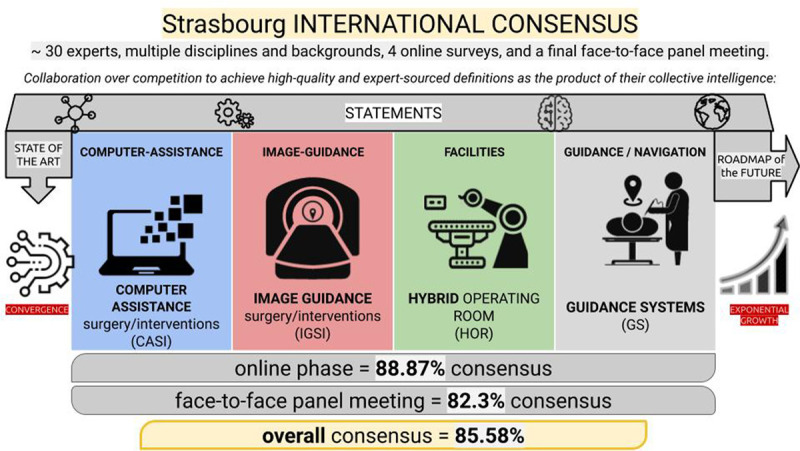
Visual abstract summarizing the results of the consensus process.

In this project, a panel of experts worked collaboratively to create high-quality definitions of computer-assisted surgery and intervention, image-guided surgery and intervention, hybrid operating room, and guidance systems and also a road map for future projects. These results might be considered an important milestone in the short history of image-guided minimally invasive surgery and intervention and also the basis of future collective efforts. There is a lot of work ahead, but collaborative efforts seeking collective intelligence seem to be an excellent approach with the potential to transform data into valuable information, significantly facilitating communication and the spread of knowledge (Fig. 6).

## Acknowledgments

The authors thank Guy Temporal and Christopher Burel for their assistance in proofreading the article and Stephanie Deming, ELS, Senior Scientific Editor, Scientific Publication Services, Research Medical Library, for her assistance with the editing of this manuscript.

## APPENDIX

### Round 1

Printable version link

### Round 2

Printable version link

### Round 3

Printable version link

### Round 4

Printable version link

## References

[1] May D Vogels E Parker D. Overall outcomes of laparoscopic-assisted ERCP after Roux-en-Y gastric bypass and sphincter of Oddi dysfunction subgroup analysis. Endosc Int Open. 2019; 7:E1276–E1280. 31579709 10.1055/a-0832-1898 PMC6773570

[2] James HJ James TW Wheeler SB. Cost-effectiveness of endoscopic ultrasound-directed transgastric ERCP compared with device-assisted and laparoscopic-assisted ERCP in patients with Roux-en-Y anatomy. Endoscopy. 2019; 51:1051–1058. 31242509 10.1055/a-0938-3918

[3] Sawas T Storm AC Bazerbachi F. An innovative technique using a percutaneously placed guidewire allows for higher success rate for ERCP compared to balloon enteroscopy assistance in Roux-en-Y gastric bypass anatomy. Surg Endosc. 2020; 34:806–813. 31139990 10.1007/s00464-019-06832-9

[4] Alabraba E Travis S Beckingham I. Percutaneous transhepatic cholangioscopy and lithotripsy in treating difficult biliary ductal stones: two case reports. World J Gastrointest Endosc. 2019; 11:298–307. 31040891 10.4253/wjge.v11.i4.298 PMC6475703

[5] Wang TJ Thompson CC Ryou M. Gastric access temporary for endoscopy (GATE): a proposed algorithm for EUS-directed transgastric ERCP in gastric bypass patients. Surg Endosc. 2019; 33:2024–2033. 30805786 10.1007/s00464-019-06715-z

[6] Wang TJ Ryou M. Evolving techniques for endoscopic retrograde cholangiopancreatography in gastric bypass patients. Curr Opin Gastroenterol. 2018; 34:444–450. 30239342 10.1097/MOG.0000000000000474

[7] Kedia P Tarnasky PR Nieto J. EUS-directed transgastric ERCP (EDGE) versus laparoscopy-assisted ERCP (LA-ERCP) for Roux-en-Y gastric bypass (RYGB) anatomy: a multicenter early comparative experience of clinical outcomes. J Clin Gastroenterol. 2019; 53:304–308. 29668560 10.1097/MCG.0000000000001037

[8] McMillan SS King M Tully MP. How to use the nominal group and Delphi techniques. Int J Clin Pharm. 2016; 38:655–662. 26846316 10.1007/s11096-016-0257-x PMC4909789

[9] Halcomb E Davidson P Hardaker L. Using the consensus development conference method in healthcare research. Nurs Res. 2008; 16:56–71. 10.7748/nr2008.10.16.1.56.c6753 19025106

[10] Trevelyan E Robinson N. Delphi methodology in health research: how to do it? Eur J Integr Med 2015; 7:423–428.

[11] Humphrey-Murto S Varpio L Wood TJ. The use of the Delphi and other consensus group methods in medical education research: a review. Acad Med. 2017; 92:1491–1498. 28678098 10.1097/ACM.0000000000001812

[12] Chia-Chien H Sandford B. The Delphi technique: making sense of consensus. Pract Assess Res Eval 2007; 12:1–8.

[13] Waggoner J Carline JD Durning SJ. Is there a consensus on consensus methodology? Descriptions and recommendations for future consensus research. Acad Med. 2016; 91:663–668. 26796090 10.1097/ACM.0000000000001092

[14] Python 3.7.4. https://www.python.org. Accessed September 30, 2019.

[15] Google forms. https://www.google.com/forms/about. Accessed September 30, 2019.

[16] Cardella JF Miller DL Cole PE; Society of Cardiovascular & Interventional Radiology. Society of Cardiovascular & Interventional Radiology position statement on radiation safety. J Vasc Interv Radiol. 2001; 12:281. 11287503 10.1016/s1051-0443(07)61905-8

[17] Miller DL Vañó E Bartal G; Cardiovascular and Interventional Radiology Society of Europe; Society of Interventional Radiology. Occupational radiation protection in interventional radiology: a joint guideline of the Cardiovascular and Interventional Radiology Society of Europe and the Society of Interventional Radiology. Cardiovasc Intervent Radiol. 2010; 33:230–239. 20020300 10.1007/s00270-009-9756-7 PMC2841268

[18] Hoag D. Apollo navigation, guidance, and control systems: a progress report. MIT Instrumentation Laboratory, 1969:1–29.

